# Enhancing mechanical performance of sandwich structures via uniform and gradient auxetic cores

**DOI:** 10.1038/s41598-026-58419-9

**Published:** 2026-06-21

**Authors:** Hasnaa W. Taha, Mohamed A. N. Shabara, Mohamed G. Elkhateeb, Tawakol A. Enab

**Affiliations:** 1 Production and Mechanical Design Engineering Department, Faculty of Engineering, Mansoura University, Mansoura, Egypt; 2 Misr Higher Institute for Engineering and Technology (MET), Mansoura, Egypt

**Keywords:** Graded auxetic structures, Re-entrant honeycomb, Sandwich structures, Energy absorption, Three-point bending, Finite element analysis., Engineering, Materials science

## Abstract

Auxetic metamaterials have attracted substantial attention as core materials for sandwich structures for advanced lightweight applications due to their unconventional deformation behavior. This investigation studies the nonlinear dynamic response of re-entrant auxetic sandwich panels with various core designs subjected to three-point bending. The examined core designs include pure auxetic and gradient variations of the unit cell wall thickness -vertically and horizontally- across the core. Acrylonitrile Butadiene Styrene (ABS) polymer is chosen as the overall material, due to its high toughness, impact resistance, good processability and suitability for additive manufacturing processes. Finite element simulations were conducted in Abaqus/CAE to evaluate the influence of auxetic core geometry on load distribution, failure behavior, and energy absorption capacity. The numerical models were validated using previously published experimental data from the literature. The results showed that the graded designs improved the distribution of loads and postponed localized failure, thus improving maximum load bearing capacity and energy absorption resulting in enhanced bending performance. The horizontal internal graded configuration exhibited the best mechanical response, achieving a 23.2% increase in maximum load capacity and a 32.8% increase in energy absorption relative to the uniform auxetic core. Furthermore, the gradient effect introduced a progressive deformation mechanism, which induced smoother force-displacement responses alongside decreased stress concentrations. These findings demonstrate that graded auxetic core architectures provide an effective approach for enhancing the mechanical performance of sandwich structures in lightweight engineering applications.

## Introduction

Sandwich structures are widely used in modern engineering applications due to their high stiffness-to-weight ratio, excellent energy absorption capability, and structural efficiency^1^. Generally, these structures consist of a lightweight core with two thin face sheets attached, the selection of core significantly influences out-of-plane stiffness, shear capacity, and energy-absorption behavior since the face-sheets bear bending and in-plane loads while the core bears shear and keeps the face sheets from buckling^2–4^. Because of these advantages, sandwich panels have been extensively utilized in aerospace, marine, automotive, and civil engineering structures^5,6^, where lightweight and high mechanical performance are required.

The microstructural design and inherent material properties of the core have an essential effect on the mechanical behavior of sandwich structures^7,8^. Since the core represents a significant portion of the component’s weight and volume, substituting dense and solid core materials with lightweight and low-density material is the key to optimizing performance and reducing the overall weight and costs of the structure^9^.

Focusing on the utilization of motion, deformation, stresses, and mechanical energy^10^, meta-materials represent a relatively new area of study. A representative unit, also referred to as a “unit cell,” is often arranged in a periodic pattern to create a mechanical meta-material. A variety of unconventional mechanical properties can be achieved^11^, including negative Poisson’s ratios (NPRs)^12^, negative stiffness^13^, negative compressibility^14^, a near-zero effective shear modulus^15^, and a high stiffness-to-weight ratio^16^, can be attained by arranging the architecture of the unit cell.

There are several core designs that have been proposed to improve the behavior of sandwich panels under various loads including honeycomb^17^, folded^18^, egg-box^19^, and lattice structures^20^.

Among the innovative core architectures proposed in recent years, auxetic materials have attracted significant attention due to their unusual deformation mechanism. Throughout the 1990’s and early 2000’s, researchers developed models of re-entrant structures^21^, rotating units^22^, Chiral structures^23^, origami-inspired patterns^24^, sliding bars systems^25^, establishing the foundation for architected auxetics^26,27^. Recent developments in additive manufacturing have further accelerated the adoption of auxetic and cellular structures in engineering applications^28–30^. Advanced manufacturing techniques enable the fabrication of complex geometries with high dimensional accuracy, allowing researchers to tailor structural performance through controlled modifications of unit-cell architecture. Several studies have demonstrated that additively manufactured polymer-based cellular structures exhibit enhanced stiffness-to-weight ratios, improved energy absorption, and superior mechanical efficiency compared with conventional lightweight designs^31–33^. Furthermore, additive manufacturing provides the flexibility required to implement functionally graded and architected cellular materials that would be difficult to fabricate using traditional manufacturing processes.

Recent studies have investigated the mechanical behavior of auxetic meta-materials and sandwich structures under various loading conditions^34–36^ confirming the strong influence of auxetic geometry on structural performance.

By optimizing deformation behavior at various scales, Auxetic materials^37^ have customizable characteristics. The advantages of these materials include low densities, high toughness, and substantial ductility, improved impact resistance, energy absorption, and enhanced mechanical stability, etc.,^38–41^.

They thereby contribute significantly to the development of next generation of engineering materials such as biomedical materials^42^, building materials^43^, aerospace components^44^, optical components^45^, and smart sensors^46,47^.

Rather than the raw material’s mechanical characteristics, the properties listed are associated with the geometrical arrangement of auxetic structures. Auxetic materials’ unique characteristics have garnered interest in a range of applications such as personal Protection^48^, Smart Textiles^49^, smart biomedical materials^50^, soft robotics^51^, sports equipment^46^, Aerospace^52^ and Automotive etc.

The uniform architecture of regular auxetic structures is defined by consistent cell size, geometry, orientation, inclination angles, and wall thickness throughout the domain. Adding new modifications to these structures involve varying geometric parameters across the structure, leading to spatially tailored mechanical properties. Using this approach, materials with customized stiffness, strength, and energy absorption characteristics can be designed^53–56^.

Functionally graded cellular structures have emerged as an effective approach for improving the mechanical performance of lightweight components. By introducing controlled variations in geometric parameters such as cell size, wall thickness, or relative density, graded architectures can redistribute stress more efficiently and delay the onset of localized failure. Previous investigations have reported significant improvements in stiffness, load-carrying capacity, and energy absorption when compared with uniform cellular configurations. These findings highlight the potential of geometric grading as a promising strategy for optimizing the performance of auxetic sandwich structures subjected to bending and impact loading conditions^57,58^.

Despite the considerable progress achieved in the development of auxetic meta-materials and functionally graded cellular structures, limited attention has been devoted to understanding the influence of gradient orientation and reinforcement placement on the flexural behavior of auxetic sandwich panels. Most existing studies have focused on uniform auxetic geometries or compression-dominated loading scenarios, while the bending response of sandwich structures incorporating graded re-entrant auxetic cores remains insufficiently investigated. In particular, the effects of horizontal and vertical grading strategies on load transfer mechanisms, stress distribution, and energy absorption performance have not been comprehensively addressed. Therefore, the present study investigates the nonlinear bending response of sandwich panels incorporating uniform and graded re-entrant auxetic cores using finite element analysis. The influence of core configuration on deformation behavior, stress distribution, maximum load capacity, and energy absorption under three-point bending conditions is systematically evaluated. The outcomes of this work provide further insight into the design of high-performance lightweight sandwich structures employing graded auxetic cores.

## Methodology

Simulations of three-point bending are performed on tailored sandwich beams where the core’s architecture is crucial in transferring shear stresses and transverse normal stresses resulting from the concentrated load. Therefore, to support these stress components, the core material must possess adequate mechanical properties^59^. In this context, to identify the optimal designs for enhanced structural performance.

This section describes the design of the sandwich structures, the material and manufacturing parameters, and the numerical modeling procedure used to investigate the bending behavior of the auxetic-core sandwich panels. The methodology was developed to ensure repeatability and to allow comparison between uniform and graded wall thickness auxetic core configurations under identical loading conditions.

### Structure design

**Fig. 1:**
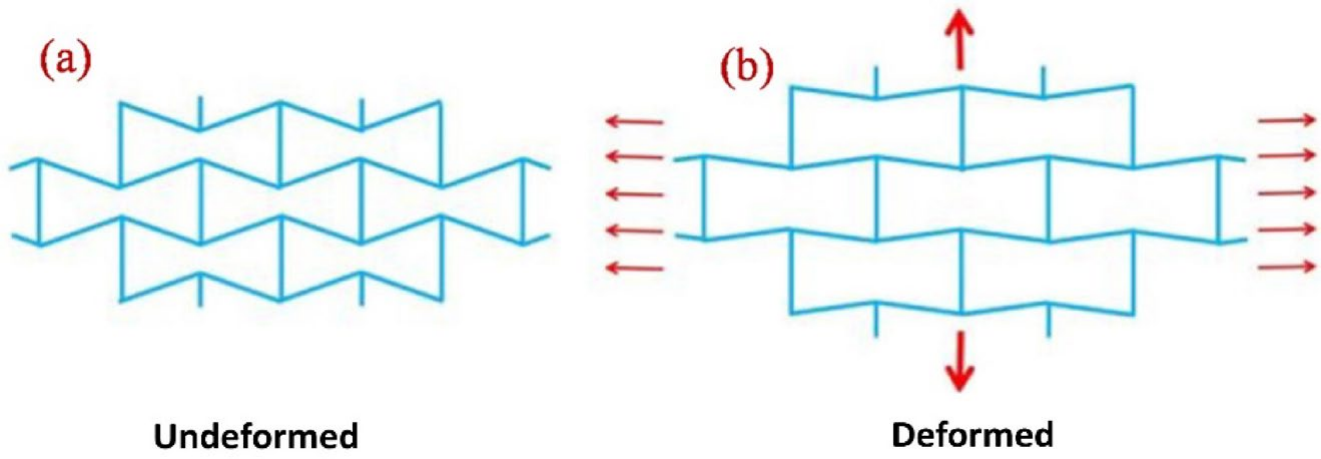
Schematic illustration of the deformation mechanism of a re-entrant auxetic structure showing lateral expansion under tensile loading^61^.

The mechanical performance of the proposed sandwich structures is governed by the deformation behavior of the re-entrant auxetic core. Unlike conventional cellular materials^60^, auxetic structures exhibit a negative Poisson’s ratio, causing them to expand laterally when subjected to tensile loading and contract laterally under compressive loading. This unique behavior results from the rotation and bending of the inclined cell ribs, which enhances load distribution, energy absorption, and resistance to localized deformation. The fundamental deformation mechanism of a re-entrant auxetic structure under tensile loading is illustrated in Fig. 1.

**Fig. 2:**
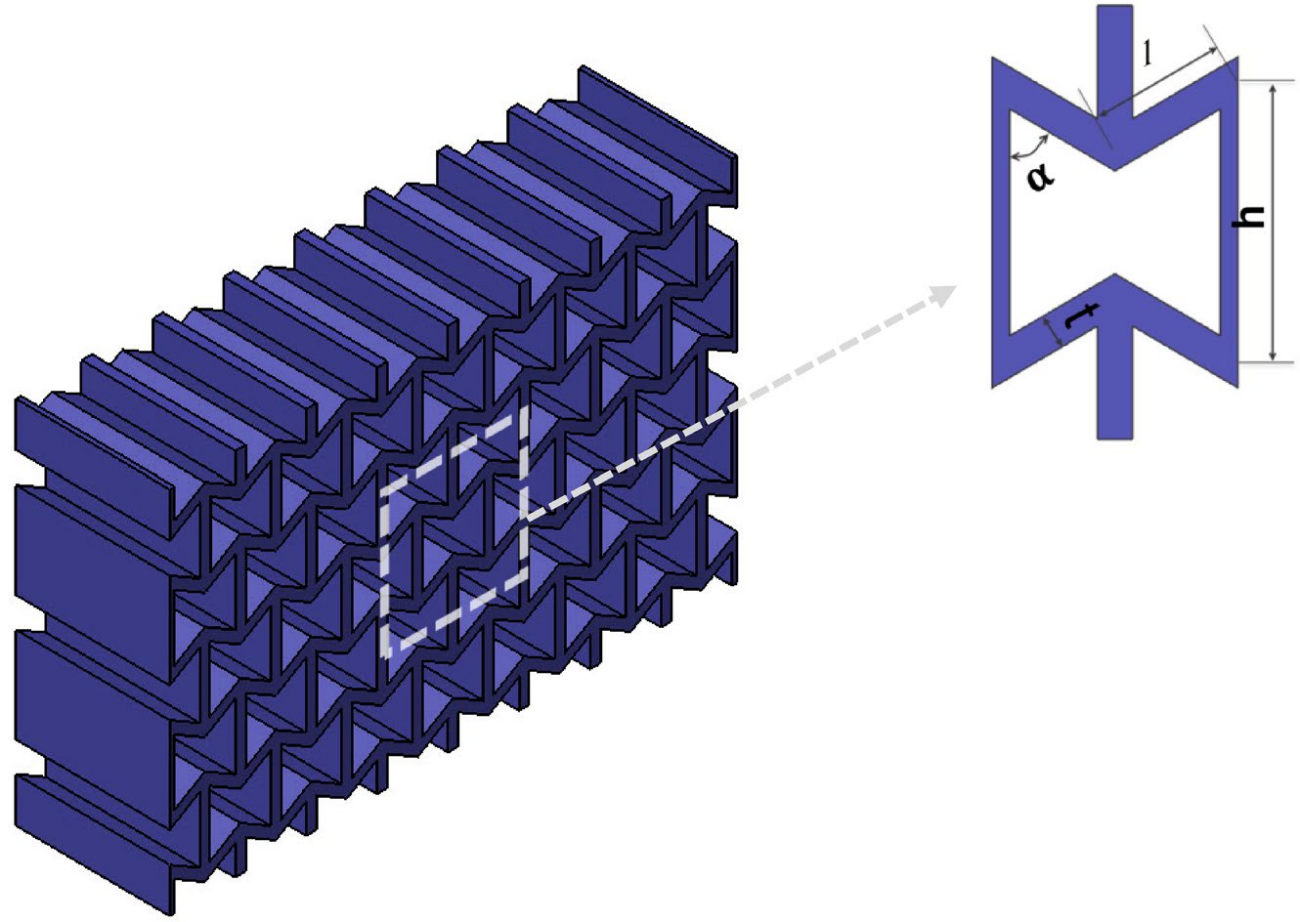
Geometry and design parameters of the re-entrant honeycomb auxetic unit cell, showing the vertical wall length (h), inclined wall length (l), wall thickness (t), and re-entrant angle (α).

In this section, the geometric features of sandwich core design, namely the re-entrant auxetic honeycomb configuration, are proposed. Fig. 2 provides the details of sandwich panels uniform core of the corresponding auxetic architecture. For the uniform unit cell, the design parameters h, l, α and t denote the length of vertical wall, the length of inclined wall, the inclination angle of the inclined walls, and the thickness of the wall, respectively. The principal geometric parameter values of the auxetic unit cell are summarized in Table 1.

**Table 1:** Geometric parameters of the auxetic unit cell.

Parameter	h (mm)	l (mm)	α (^o^)	t (mm)
Value	8	4	60	1

**Fig. 3:**
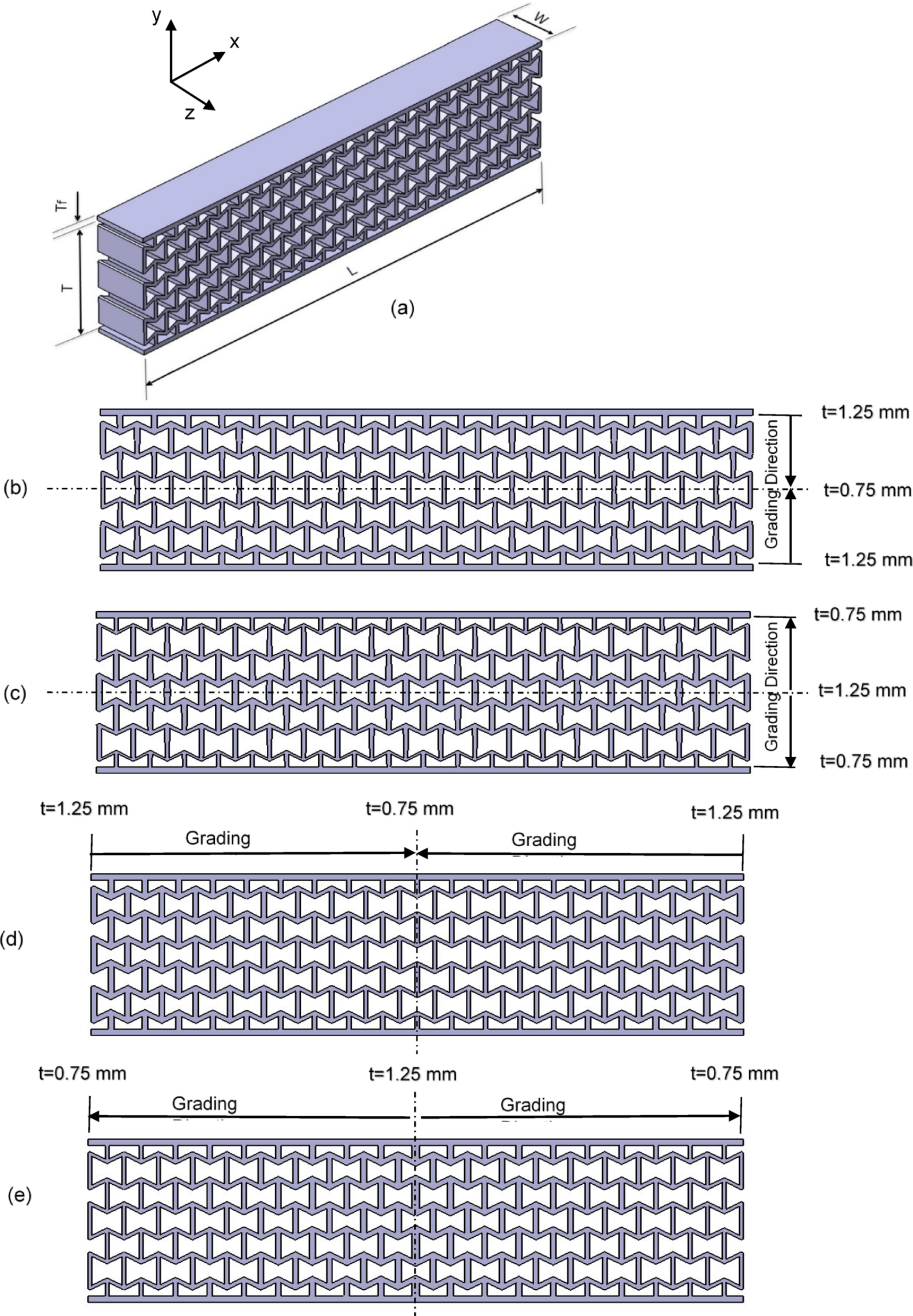
Geometrical configurations of the investigated auxetic sandwich panels: (**a**) uniform core, (**b**) vertical external gradient (VEG), (**c**) vertical internal gradient (VIG), (**d**) horizontal external gradient (HEG), and (**e**) horizontal internal gradient (HIG).

The core specimen design adheres to ASTM C393^62^ requirements for evaluating the flexural behavior of sandwich structures. Fig. 3 shows a CAD model of 5 types of auxetic re-entrant honeycomb core structures which are uniform, vertical external graded (VEG), vertical internal graded (VIG), horizontal external graded (HEG) and horizontal internal graded (HIG) re-entrant structure. All specimens share the same global dimensions, all the auxetic sandwich models consist of 19-unit cells along x-direction and 3-unit cells along y-direction, while the graded configurations differ solely in the wall thickness gradient. The figure also represents the direction of wall thickness grading -vertically and horizontally- from 1.5 mm to 0.75 mm and vice versa, to ensure the same mass of the five types of uniform and graded sandwich structures. To ensure consistency across all sandwich beam specimens, an equal number of unit cells was maintained, resulting in an overall beam length (L) of approximately 132 ± 1 mm. The three-point bending test simulation was conducted with a span length of 90 mm. Each specimen had a depth (W) of 15 mm, and its overall thickness (T) is 36 mm. The applied load was introduced via a centrally positioned punch with a radius of 15 mm, while two identical fixed supported rollers of the same radius were used at each end. To ensure uniform load distribution across the structures, two face sheets—each with a thickness (T_f_) 1.5 mm—were integrated at the top and bottom of all specimens.

### Materials

**Fig. 4:**
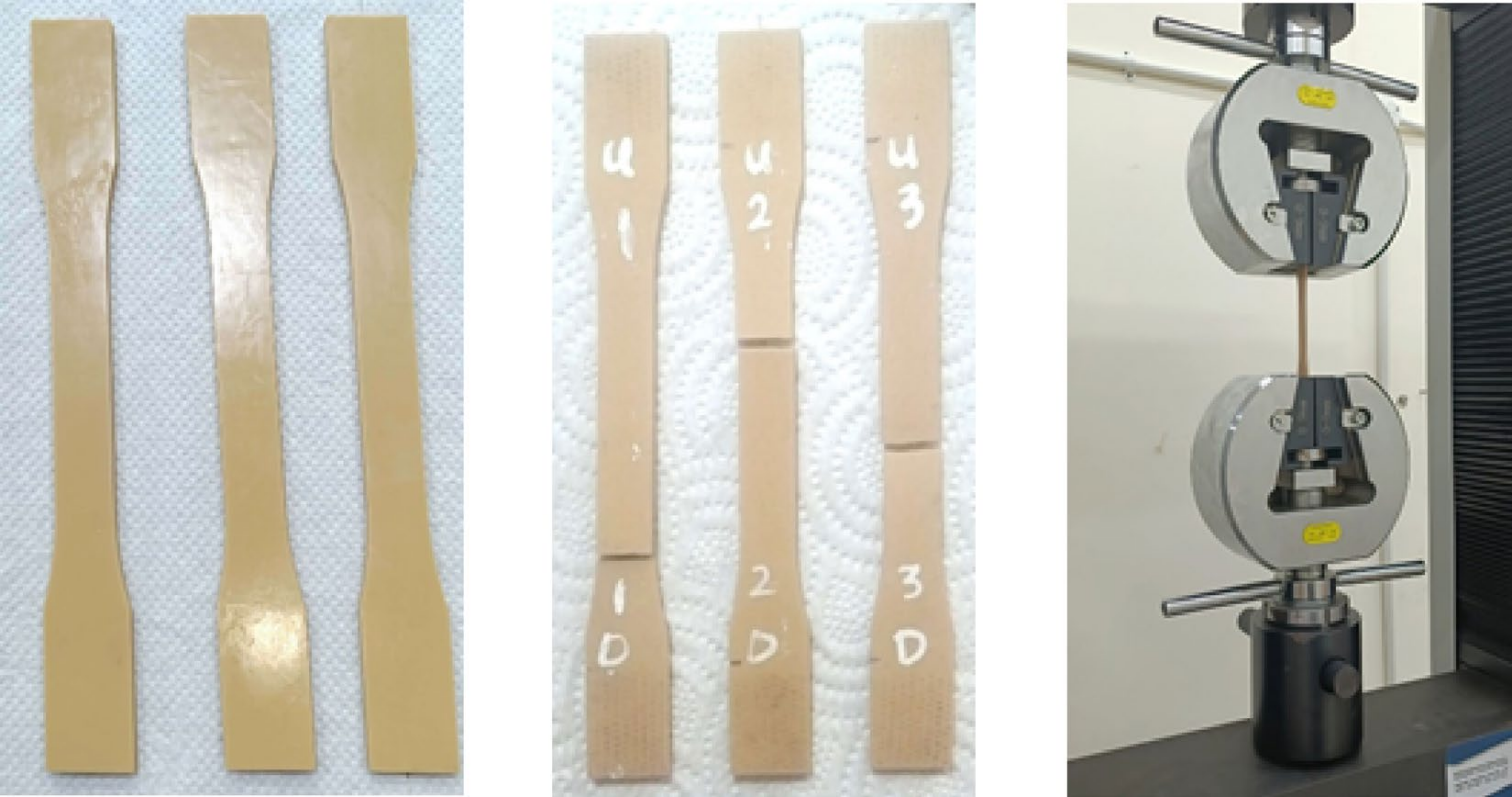
3D printed dog-bone coupons subjected to uniaxial Loading.

The sandwich structure’s mechanical behavior depends strongly on the constitutive response of the base material used for both the face sheets and the auxetic core, so that the base material used should be characterized. Acrylonitrile Butadiene Styrene (ABS) polymer was chosen for this investigation because of its extensive application in additive manufacturing and its advantageous blend of ductility, toughness, and stiffness. It also exhibits sufficient ductility to accommodate the deformation mechanisms typically observed in auxetic cellular structures^63^. The specimens were 3D printed using Phrozen Sonic Mighty 4 K LCD printer. The printing parameters employed during specimen fabrication included a layer height of 0.050 mm, an exposure time of 3 s, bottom exposure time of 35 s, and a 100% infill density. Uniaxial tensile tests were conducted on three identical dog-bone test coupons, according to ASTM-D638 standard^64^, using a WDW-600 computer controlled electronic universal testing machine (UTM) equipped with a 5 KN load cell. During testing, the specimens were securely mounted in the grips of the UTM, and tensile loading was applied at a constant crosshead speed of 2 mm/min until failure occurred. Figure 4 displays the dog-bone shaped specimens before and after testing and the specimens positioned for uniaxial tensile testing.

**Fig. 5:**
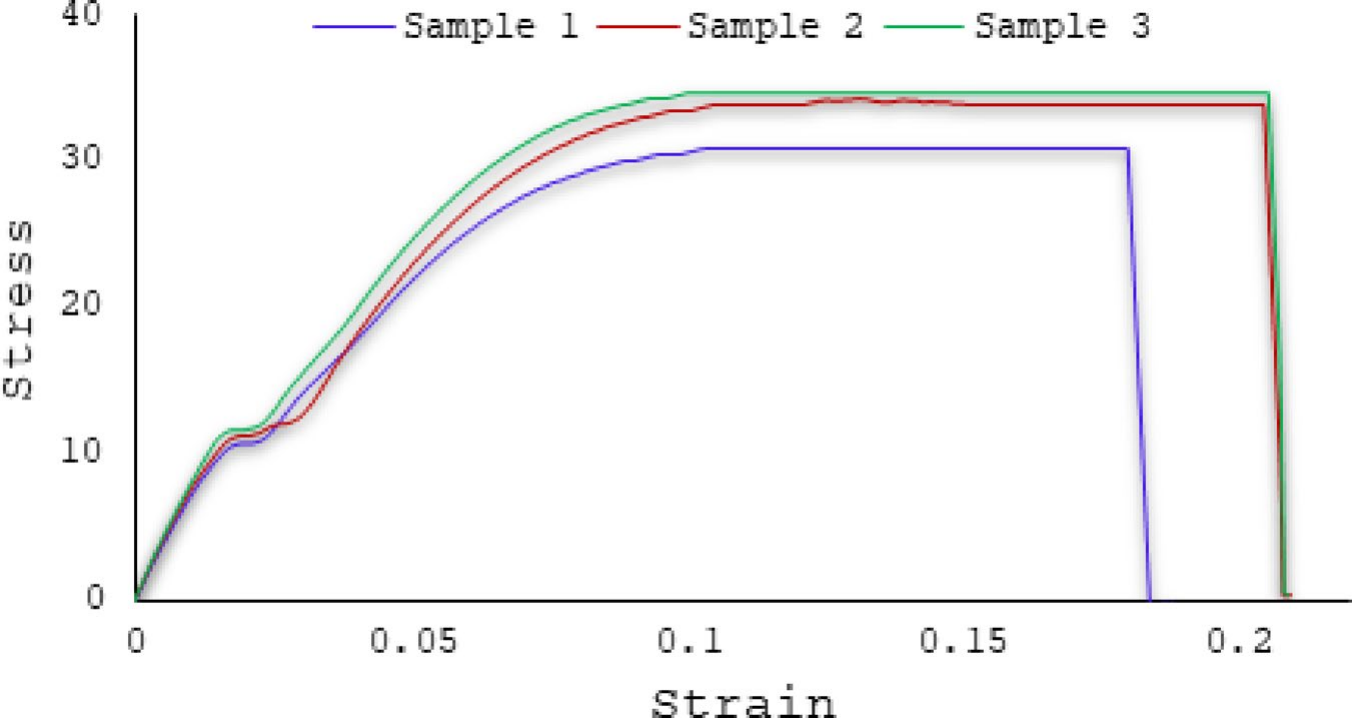
True stress-strain curves of 3D printed ABS dog-bone coupons under tensile load used for material characterization in the numerical simulations.

The experimental data are analyzed with Origin software to calculate the mechanical properties of the specimens, The true stress–strain response of the ABS material, is illustrated in Fig. 5. Table 2 summarizes the corresponding average mechanical properties derived from the test data- Young’s modulus, yield stress- while density and Poisson’s ratio were obtained from the material datasheet provided by the manufacturer and previously reported literature values^35,65^. These mechanical properties were employed to define the material behavior in the finite element simulations. The use of experimentally measured properties ensures that the numerical model accurately captures the mechanical response under applied loading conditions. Considering that the auxetic response discussed in this study refers to the effective structural behavior of the cellular geometry rather than the intrinsic Poisson’s ratio of the base material.

**Table 2:** Material properties of the base material used in the analysis.

Material	Young’s modulus (MPa)	Poisson’s ratio^65^	Yield stress (MPa)	Density (g/cm^3^)
ABS	1900 ± 10	0.35	30 ± 1	1.05

### Numerical modelling

**Fig. 6:**
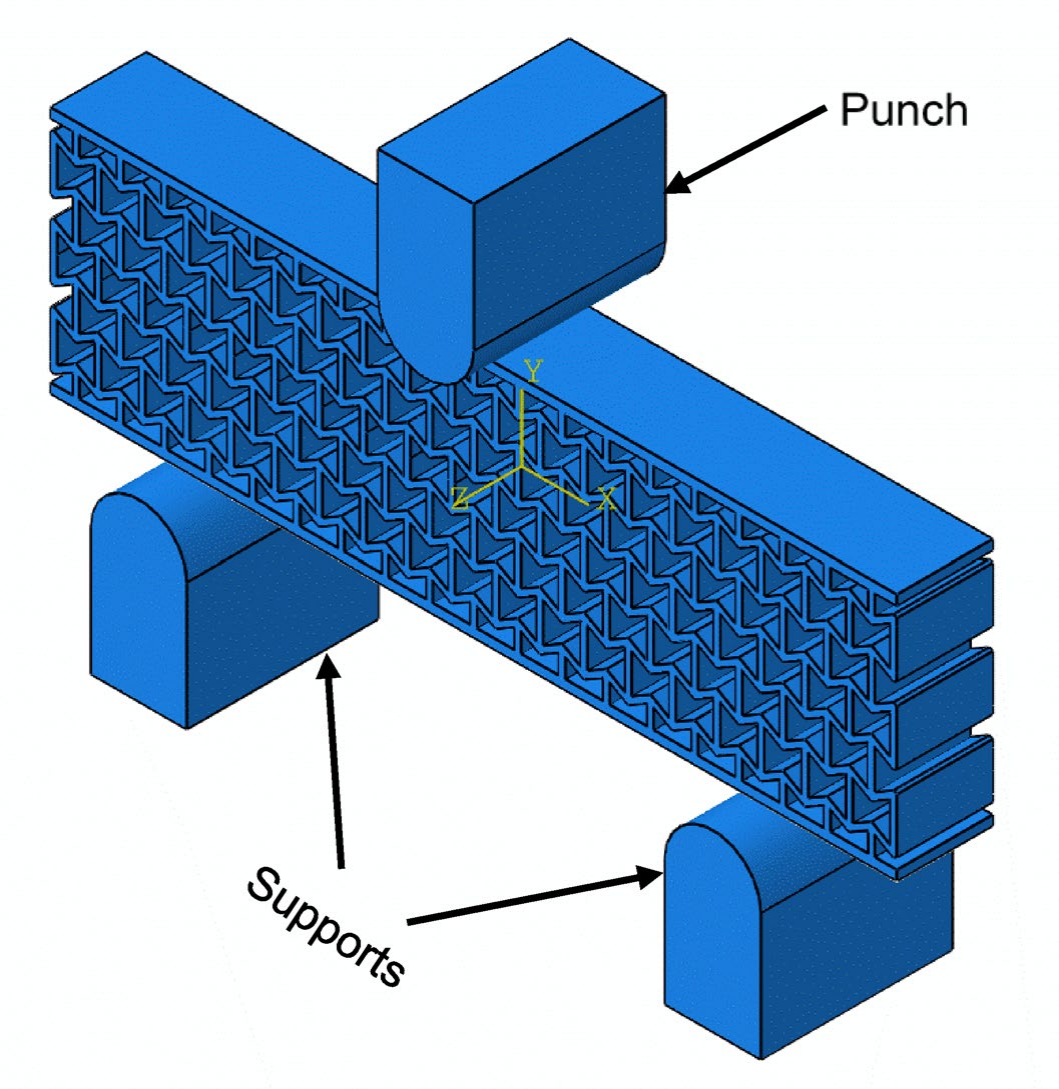
Three-point bending test setup showing the upper anvil (Punch), the lower supports, and auxetic sandwich specimen configuration.

Quasi-static three-point bending simulation was performed using the finite element software package ABAQUS. Explicit/Dynamic models are conducted to accurately capture the nonlinear mechanical response (due to large deformations) of the structure under flexural loading. The geometric model of the sandwich panel and support fixtures was initially designed in a computer-aided design (CAD) software and subsequently imported into Abaqus in a suitable format (e.g., STEP or Parasolid). Upon import, the assembly was configured to reflect the physical test setup, with rigid bodies representing the upper anvil (Punch) and the lower supports as illustrated in Fig. 6.

The material properties of the sandwich panel components were defined based on experimental data obtained from uniaxial tensile testing as previously mentioned in Sect. 2.2. A perfectly elastic–plastic material model was employed; the elastic response was characterized in Table 1 and plastic behavior specified through true stress–strain data. Rigid body constraints were applied to the lower anvils to fully restrict translational and rotational degrees of freedom, simulating fixed supports. The punch was defined as a discrete rigid body and subjected to a prescribed vertical displacement to replicate the bending load with a reference point at the center.

Surface interactions were modeled using a general contact formulation (ALL WITH SELF), with normal behavior and tangential behavior with the penalty-based friction coefficient of 0.25 - based on typical values reported for polymer–metal interfaces in mechanical contact studies^66,67^ – this value has been widely used in numerical simulations of polymer-based structural systems to account for realistic contact behavior between the punch and the specimen. To guarantee numerical stability and precision in capturing stress gradients, the face sheets and core mesh were created using linear eight-node brick elements (C3D8I), hexahedral elements with a sweep meshing technique and an element size of 0.5 mm, which was selected based on mesh convergence studies reported in previous investigations of auxetic cellular structures and sandwich panels^68,69^. These studies demonstrated that this mesh resolution is sufficient to accurately capture deformation behavior and stress distribution. Considering the geometric features of the re-entrant auxetic core and the need to properly represent cell wall deformation during bending, the selected mesh size provides a good balance between numerical accuracy and computational efficiency. R3D4 (4-node rigid quadrilateral) elements were used to discretize the punch and supports, which were modeled as rigid shells with designated reference points and show no deformation under applied loads.

Each auxetic core sandwich structural model has a different number of nodes and elements. For example, there were 390,445 nodes and 273,900 elements in the uniform structure’s finite element model. Table 3 summarizes the total elements and nodes for each structure.

**Table 3:** The finite element models’ total number of elements and nodes.

Structure	Number of elements	Number of nodes
Uniform	273,900	390,445
Vertical external graded	267,870	381,052
Vertical internal graded	264,480	383,098
Horizontal external graded	273,330	388,740
Horizontal internal graded	273,780	388,957

**Fig. 7:**
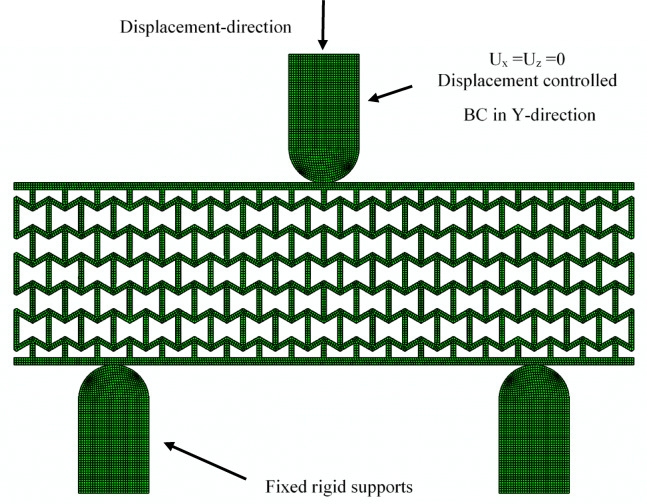
Finite element model showing mesh discretization, contact interactions, boundary conditions, and loading arrangement used in the dynamic explicit analysis.

An explicit dynamic step, which is appropriate for managing highly nonlinear systems with significant deformations and intricate contact conditions, was used to run the simulation. To replicate the experimental three-point bending test configuration and guarantee convergence and stability of the numerical solution, boundary conditions, loading rates, and time increment parameters were carefully chosen. The meshed model with the specified boundary conditions is shown in Fig. 7.

## Results and discussion

### Model verification

**Fig. 8:**
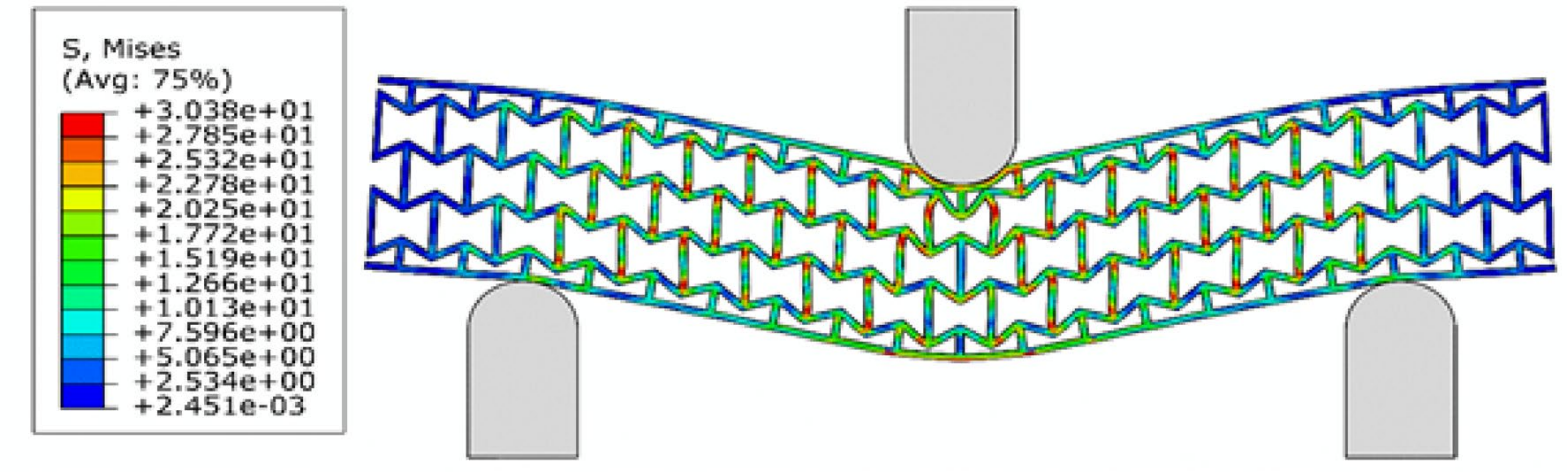
Finite element models adopted from the literature for numerical validation of the developed model.

To ensure the reliability of the developed finite element model, the developed models were validated against experimental and numerical results reported in the literature^69^ for auxetic sandwich structures subjected to three-point bending. Figure 8 shows the developed FE models that are made with identical geometry, same material, same boundary conditions for certain cases in the literature. The validation was performed by comparing the load–displacement response obtained from the numerical simulations with the experimental and numerical curves in the literature.

**Fig. 9:**
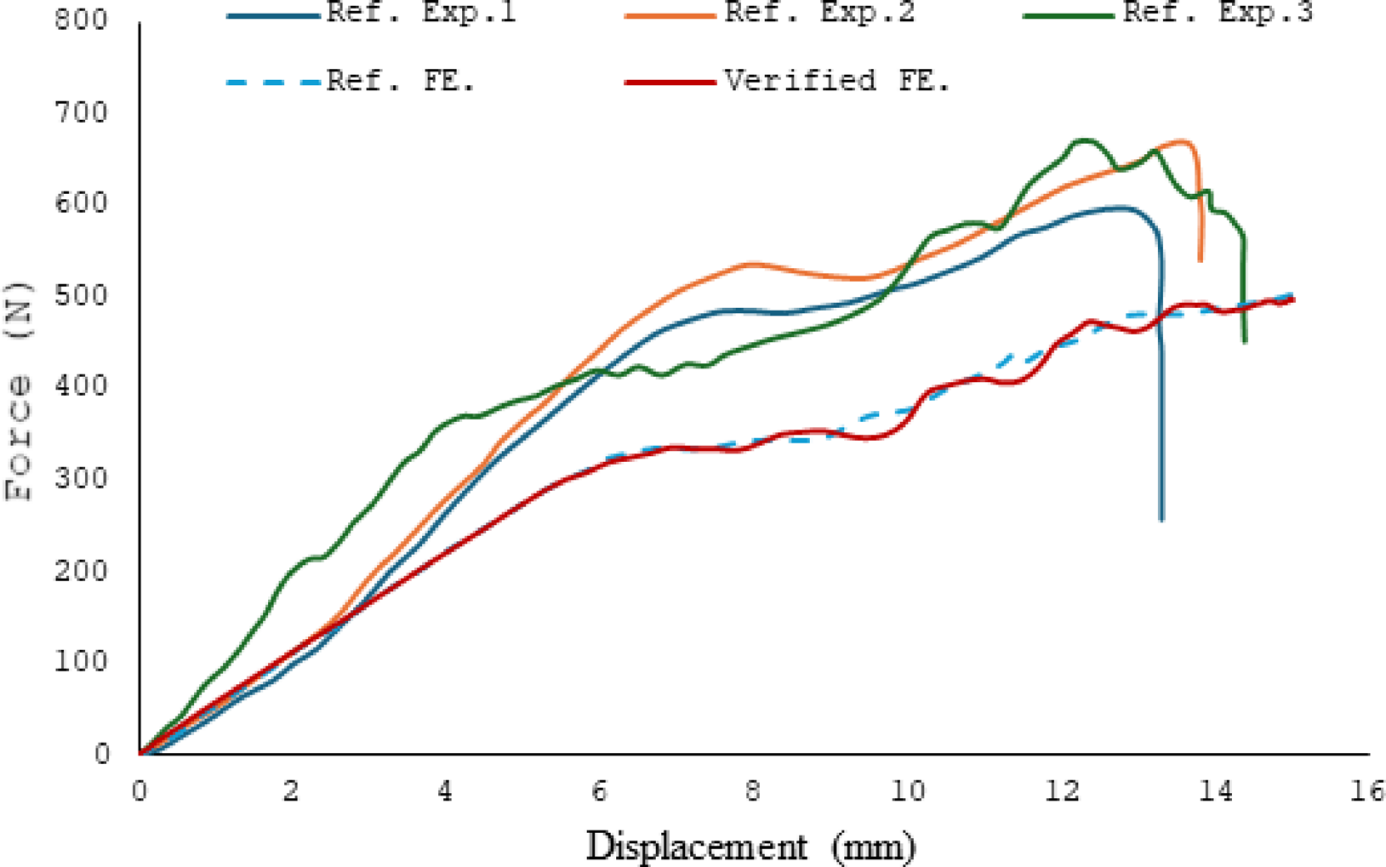
Comparison of load–displacement responses obtained from the developed finite element model and literature results used for model validation.

Figure 9 shows the comparison between experimental and numerical results in literature and the FE models of this study. As shown in the figure, the numerical results follow the same trend as the experimental data throughout the loading process. In the initial stage of deformation (0–6 mm displacement), the predicted response shows excellent agreement with the experimental curves. As the displacement increases, the load gradually rises due to progressive deformation of the auxetic core structure.

The maximum load predicted by the numerical model is approximately 500 N, which lies within the range of the experimental results (approximately 590–670 N). The difference between the numerical prediction and the experimental values can be attributed to several factors, including manufacturing imperfections, material variability, and simplifications in the numerical model such as idealized geometry and uniform material properties.

Overall, the finite element model demonstrates a good level of agreement with the experimental results in terms of deformation trend and load response. Therefore, the developed model is considered sufficiently accurate to investigate the influence of auxetic core configurations on the bending performance of sandwich panels.

### Results and discussion of quasi‑static simulation

This study investigates the mechanical behavior of ABS-based re-entrant auxetic structures under quasi-static three-point bending, with particular focus on how wall thickness gradients influence load- bearing and energy absorption capabilities. The nonlinear explicit finite element simulations capture both the localized stress response and the global structural deformation, yielding key insights into gradient-driven performance variations.

**Fig. 10:**
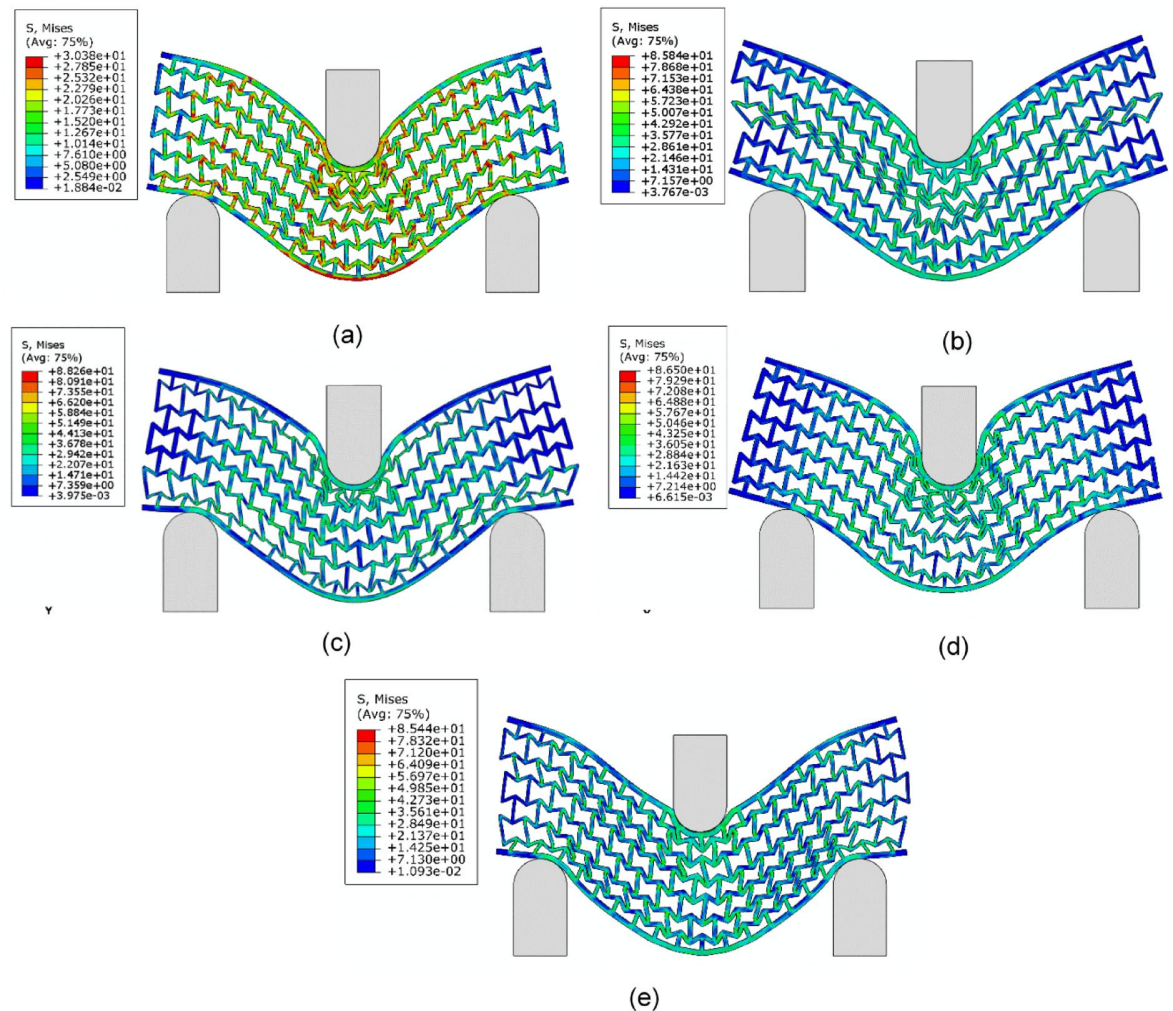
Von Mises stress distribution of the uniform and graded-thickness auxetic core sandwich panels under quasi-static 3-point bending load: (**a**) Uniform, (**b**) VEG, (**c**) VIG, (**d**) HEG, and (**e**) HIG.

As illustrated in Fig. 10 (von Mises stress contours), the deformation of the auxetic structures under the centrally applied load reveals a highly localized stress concentration beneath the loading punch, with stresses propagating symmetrically along the re-entrant arms of the honeycomb cells. Auxetic geometry contributes to a unique deformation mechanism that allows lateral expansion under compression, effectively distributing stress across a broader area. This mitigates the severity of stress peaks and delays the onset of material failure.

**Fig. 11:**
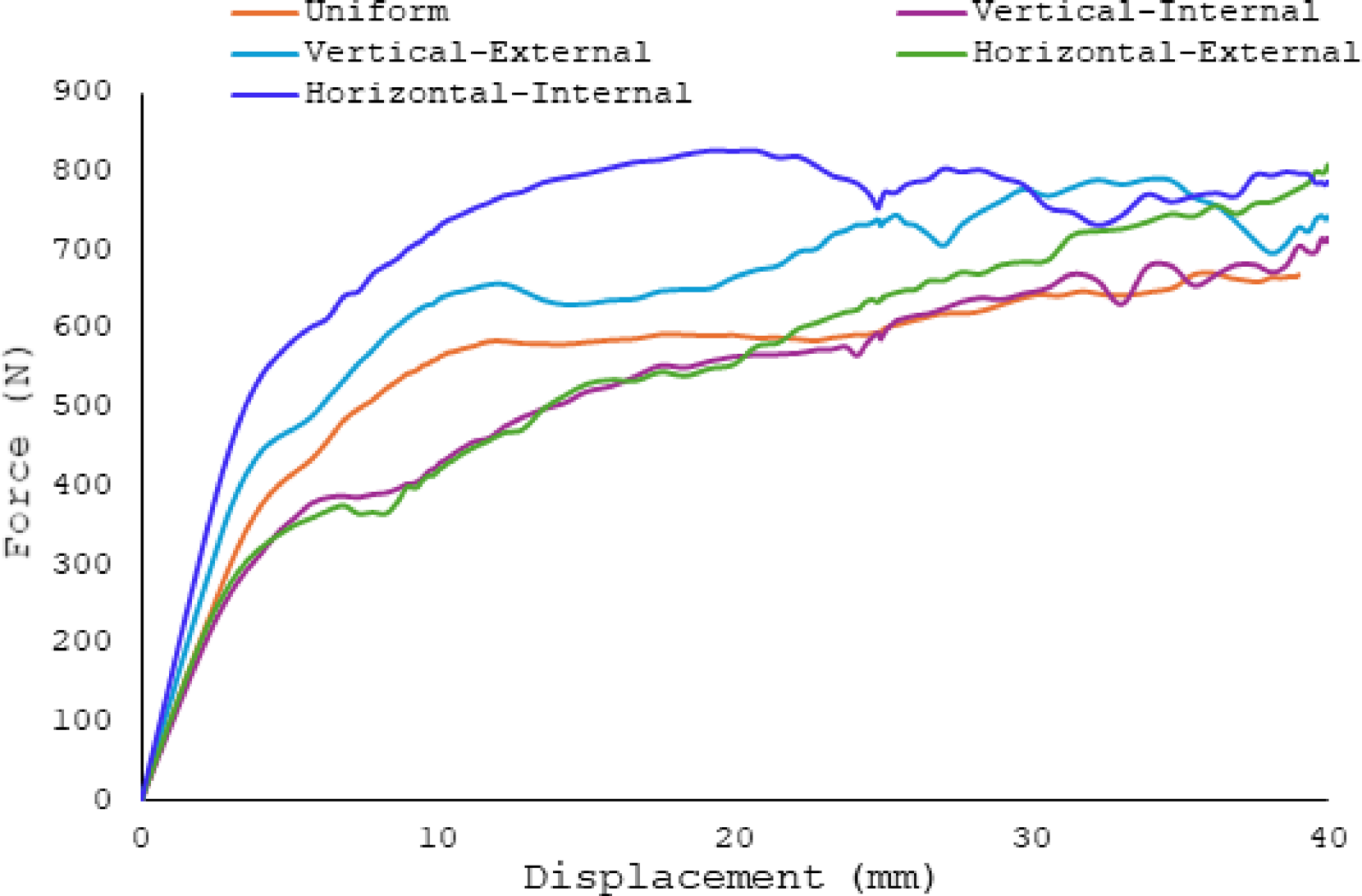
Load–displacement curves of the investigated auxetic sandwich panels under three-point bending, showing the influence of wall-thickness gradient orientation and reinforcement location on the mechanical response.

Figure 11 shows the force-displacement curves for five configurations: Uniform, Vertical-Internal, Vertical-External, Horizontal-External, and Horizontal-Internal auxetic sandwich panels. All structures display an initial linear response followed by a nonlinear plateau and post-peak softening, characteristic of plastic deformation in ABS under bending.

Horizontal-Internal Gradient configuration exhibits the highest peak force, reaching ~ 850 N, and sustains high force over a wide displacement range (> 35 mm), indicating superior energy absorption and resistance to failure. The strategic placement of material along the internal surfaces—especially in the horizontal direction—supports gradual load transfer and mitigates premature collapse by distributing deformation across the width of the structure.

While the Vertical-External Gradient variant shows the second-best performance, peaking near 750 N. The vertical thickening of external walls enhances stiffness near the tensile and compressive zones, improving resistance to bending moments. However, its load plateau is slightly shorter and less stable compared to the horizontal-internal case.

Although Horizontal-External Gradient configuration outperforms the uniform and vertical-internal designs, its force plateau is more irregular, with visible fluctuations. This suggests localized instabilities or progressive cell wall failures despite better engagement of lateral structural elements.

The baseline Uniform structure shows lower peak force (~ 600 N) and a relatively flat response beyond 15 mm displacement. The absence of gradient-based reinforcement leads to earlier localized yielding, reduced ductility, and suboptimal energy absorption.

Vertical-Internal Gradient is the least effective gradient strategy, even slightly underperforming the uniform case in the nonlinear regime. Concentrating material near the neutral axis provides minimal resistance to bending stresses, as tensile and compressive zones dominate three-point bending behavior.

Comparative performance clearly demonstrates that gradient orientation (horizontal vs. vertical) and material placement (internal vs. external) significantly influence structural mechanics:

- Horizontal gradients, especially internal, promote distributed deformation across the cell rows, enabling the structure to engage more material during loading and avoid sudden failures.
- External reinforcement, when applied vertically, enhances bending strength but may lead to stress localization unless complemented with horizontal variation.
- Internal vertical reinforcement offers limited benefits, highlighting the importance of aligning reinforcement direction with principal stress flow.

These findings align with classical beam theory, where maximum stress occurs at the outermost fibers during bending, making external reinforcement more effective^70^.

Table 4 summarizes the values of energy absorption and maximum load. The results clearly indicate that the internal cellular configuration substantially influences the overall performance of the sandwich panels and plays a critical role in determining their bending properties. Notably, the horizontal-internal graded core exhibited superior bending behavior, achieving a significantly higher energy absorption of 26.7 J.

**Table 4:** Energy absorption and maximum load values for sandwich panels with diferent core topologies.

Structure	Energy absorption (J)	Maximum load(*N*)
Uniform	20.0847	671.498
VEG	23.8997	790.73
VIG	19.3383	716.269
HEG	20.404	808.444
HIG	26.6646	827.186

The simulation results suggest that horizontal-internal wall thickness grading is the most promising design strategy for enhancing auxetic structural performance under bending. This approach balances stiffness, strength, and ductility, making it ideal for applications requiring high energy absorption such as protective gear, biomedical support, and lightweight impact-resistant components.

The ability to tailor wall thickness using gradient-based design opens up significant possibilities for functional grading in metamaterial structures, where material usage can be optimized locally based on performance requirements.

## Conclusion

This study examined the bending performance of sandwich structures incorporating uniform and graded re-entrant auxetic cores through finite element analysis under quasi-static three-point bending conditions. The results obtained demonstrate that the introduction of wall-thickness grading has a significant influence on the structural response of auxetic sandwich panels. The main findings of this study are summarized as follows:

- Graded auxetic core designs improved stress distribution within the sandwich structure and delayed the onset of localized deformation and failure.
- Among the investigated configurations, the horizontal internal gradient (HIG) structure exhibited the best mechanical performance, achieving the highest load-bearing capacity and energy absorption.
- The HIG configuration increased the maximum load by 23.2% and enhanced the energy absorption capacity by 32.8% compared with the uniform auxetic structure.
- The results indicate that the orientation of the gradient plays a crucial role in the mechanical behavior of auxetic sandwich panels, with horizontal grading providing superior performance compared with vertical grading.

Overall, the findings confirm that geometric grading of re-entrant auxetic cores is an effective approach for improving the bending resistance and energy absorption capability of lightweight sandwich structures.

Future work will focus on the experimental validation of the proposed graded auxetic designs through additive manufacturing and mechanical testing to further verify the numerical findings. In addition, further studies may investigate alternative gradient strategies, different auxetic unit-cell geometries, and multi-material configurations in order to enhance energy absorption and structural performance for advanced lightweight engineering applications.

## Data Availability

All data generated or analyzed during this study are included in this published article.
